# The Friction Properties of Firebrat Scales

**DOI:** 10.3390/biomimetics4010002

**Published:** 2019-01-04

**Authors:** Yuji Hirai, Naoto Okuda, Naoki Saito, Takahiro Ogawa, Ryuichiro Machida, Shûhei Nomura, Masahiro Ôhara, Miki Haseyama, Masatsugu Shimomura

**Affiliations:** 1 Chitose Institute of Science and Technology, Bibi758-65, Chitose 066-8655, Hokkaido, Japan; hi7liter1fire0b@gmail.com (N.O.); m-shimom@photon.chitose.ac.jp (M.S.); 2 Graduate School of Information Science and Technology, Hokkaido University, N-14, W-9, Kita-ku, Sapporo 060-0814, Hokkaido, Japan; saito@lmd.ist.hokudai.ac.jp (N.S.); ogawa@lmd.ist.hokudai.ac.jp (T.O.); miki@ist.hokudai.ac.jp (M.H.); 3 Sugadaira Research Station, Mountain Science Center, University of Tsukuba, Sugadaira Kogen, Ueda 386-2204, Nagano, Japan; machida@sugadaira.tsukuba.ac.jp; 4 Department of Zoology, National Museum of Nature and Science Amakubo 4-1-1, Tsukuba 305-0005, Ibaraki, Japan; nomura@kahaku.go.jp; 5 The Hokkaido University Museum, N 10, W8, Sapporo 060-0810, Hokkaido, Japan; ohara@museum.hokudai.ac.jp

**Keywords:** firebrat, friction, AFM, colloidal probe, scale, microstructure

## Abstract

Friction is an important subject for sustainability due to problems that are associated with energy loss. In recent years, micro- and nanostructured surfaces have attracted much attention to reduce friction; however, suitable structures are still under consideration. Many functional surfaces are present in nature, such as the friction reduction surfaces of snake skins. In this study, we focused on firebrats, *Thermobia domestica*, which temporary live in narrow spaces, such as piled papers, so their body surface (integument) is frequently in contact with surrounding substrates. We speculate that, in addition to optical, cleaning effects, protection against desiccation and enemies, their body surface may be also adapted to reduce friction. To investigate the functional effects of the firebrat scales, firebrat surfaces were observed using a field-emission scanning electron microscope (FE-SEM) and a colloidal probe atomic force microscope (AFM). Results of surface observations by FE-SEM revealed that adult firebrats are entirely covered with scales, whose surfaces have microgroove structures. Scale groove wavelengths around the firebrat’s head are almost uniform within a scale but they vary between scales. At the level of single scales, AFM friction force measurements revealed that the firebrat scale reduces friction by decreasing the contact area between scales and a colloidal probe. The heterogeneity of the scales’ groove wavelengths suggests that it is difficult to fix the whole body on critical rough surfaces and may result in a “fail-safe” mechanism.

## 1. Introduction

Friction is an important issue that is related to saving energy and preventing the wear of parts in a wide variety of fields, such as the automobile industry [1] and medicine [2]. Nowadays, lubricants are mainly used for reducing friction forces; however, other technologies are now in demand, because lubricants generally lead to pollution of the environment [3]. In recent years, surface nano- and microstructures have attracted attention to reduce friction force [4,5,6]. Although there are a lot of reports on friction reduction by nano- and microstructures, the most effective textures remain under consideration, as friction is a complex phenomenon that is affected by atomic-level structures [7,8]. In nature, the surfaces of living organisms have been adapted to their environments during their evolution process [9,10,11], and various functions have been generated by surface nano- and microstructures: the superhydrophobic, self-cleaning surfaces of lotus leaves [12]; anti-reflection surfaces of moth eyes [13,14]; drag reduction surfaces of shark skins [15,16]; among others [9,17]. Some friction control surfaces of animals have been also reported. The snake reduces friction force on its body in order to decrease damage by using surface microdimple structures [18]. The grasshopper has hexagonal microstructures on its foot to controls stick-slip motion on dry surfaces, and to maintain friction force on wet surfaces for preventing a hydroplaning effect [19]. Here, we focused on the firebrat, *Thermobia domestica* [20,21,22,23,24], a type of primitively wingless insect belonging to the order *Zygentoma*. Because firebrats live in narrow spaces, such as openings of bookshelf, so that the body surface is frequently in contact, worn by surrounding surfaces. We speculate that their body surface might have evolved to reduce friction and to protect them from wear. Adult firebrats are known to bear scales, so that we present surface observations and friction force measurements of firebrat scale surfaces by a field-emission scanning electron microscopy (FE-SEM) and a colloidal probe atomic force microscopy (AFM) [25,26,27] as part of their surface frictional properties. We expect that these results would contribute to create a general design for a surface texturing of industrial components, such as a gear, gasket, etc.

## 2. Materials and Methods

### 2.1. Animal Materials

Firebrats, *Thermobia domestica* Packard (Zygentoma, Lepismatidae)*,* of all stages were commercially purchased (Aqua Sphere, Nagoya, Japan) reared and maintained in a commercially available plastic case (H 14 cm × W 20 cm × L 30 cm) with cardboard in an artificial climate chamber (LH-60PFP, Nippon Medical & Chemical Instruments Co., Ltd., Osaka, Japan) at a temperature of 35 °C during daytime and 30 °C at nighttime, at a relative humidity of 90%. Firebrats were fed putting water bottles covered by a gauze and common golden fish fodder. In addition, fresh, living adults were collected for surface observations, Fourier transform infrared spectroscopy (FTIR) measurements, water contact angle, and friction measurements. In order to avoid injury, firebrats were kept in a refrigerator in order to immobilize them and were sacrificed using ethyl acetate (Fujifilm Wako Pure Chemical Industries, Ltd., Osaka, Japan) vapor before using them for the experiments. 

### 2.2. Observations of Firebrats’ Surface

Surface observations of adult firebrats were carried out by a laser microscope (OLS4000, Olympus Corporation, Tokyo, Japan), and FE-SEM (5 kV, 90 µA, JSM-7800F, JEOL, Tokyo, Japan) after sputtering with Pt to a thickness of about 6 nm (30 mA, 80 s, JEC-3000FC, JEOL, Tokyo, Japan). Thereafter, groove wavelengths of firebrat body scale surfaces were analyzed by the methods of periodicity detection using autocorrelation [28]. By taking the SEM images of scales as two-dimensional signals, their autocorrelation could be calculated, where autocorrelation is a mathematical representation of the degree of similarity between a given signal and a spatially lagged version of itself. The autocorrelation has peaks at the integer time of the wavelengths of the target two-dimensional signal. Thus, by finding the first peak from the autocorrelation, the groove wavelength could be detected from the target scales. We call this groove wavelength detection method “periodicity detection using autocorrelation”. Note that totally 298 observable scales from an adult male firebrat were used for calculating the groove wavelengths (target scale numbers of each region are: 40 (head); 100 (pronotum); 80 (metanotum); 35 (8th abdominal tergum); and 43 (9th abdominal tergum).

### 2.3. Chemical and Wettability Analysis of Firebrat Scale Surface

Surface chemistry and wettability of firebrat scales before and after washing with chloroform (Fujifilm Wako Pure Chemical Industries, Ltd., Osaka, Japan) were measured by Fourier transform infrared spectroscopy (FTIR; PerkinElmer, Spotlight400 Spectrum100, Waltham, MA, USA) and water contact angle analyzer (FAMAS, Drop Master 500, Kyowa Interface Science, Saitama, Japan). FTIR spectra were collected from firebrat back surfaces placed on a glass slide before and after chloroform (CHCl_3_) treatment in the reflectance mode at a spectral resolution of 4 cm^−1^ in the frequency region from 4000 to 680 cm^−1^ by a linear array mercury-cadmium-telluride (MCT) focal plane array detector. The volume of the ultrapure water (25 °C, 18.2 Ω, Milli-Q Advantage, Merck KGaA, Darmstadt, Germany), was 2.0 µL for measuring water contact angles.

### 2.4. Friction Measurements on a Body Surface

Surface topology of scales was measured by AFM (AFM5100N, Hitachi High-Technologies Corporation, Tokyo, Japan) with conventional needle-type probe (apex curvature radius of 8 nm, FMR-20, NanoWorld, Neuchâtel, Switzerland). Surface friction forces were measured in two ways; i.e., to measure the friction forces of the scales, including the scale boundaries, a whole sacrificed firebrat was fixed on a silicon substrate using carbon adhesive tape to keep the measuring area horizontal (see Supplementary Figure S1). Subsequently, friction forces of the firebrat surface were directly measured by AFM with the conventional needle-type and colloidal probes with diameters of 5, 10, and 20 µm (CP-CONT-BSG-A, CP-CONT-BSG-B, and CP-CONT-BSG-C, sQube, Bickenbach, Germany. Information of cantilevers is shown in Supplementary Table S2a. The scanning area was 50 µm × 50 µm and the scan rate was 0.3 Hz. The scanning was performed from the scale base to the apex, from the scale apex to the base, and along lateral directions. 

### 2.5. Friction Measurements within a Scale

For detailed analysis of the relationship between scale surface structures and the dimensions of rubbed objects (indenters), a single scale was taken from a firebrat and was fixed on a silicon substrate using poly(vinyl alcohol) (Wako Pure Chemical Industries, Ltd., Osaka, Japan) as an adhesive. Afterwards, friction forces were measured by AFM with needle and colloidal probes with diameters of 2.0, 3.5, and 6.6 µm (CP-CONT-SIO-A, CP-CONT-SIO-B, and CP-CONT-SIO-C, sQube, Germany. Information of cantilevers is shown in Supplementary Table S2b. The scanning area was 15 µm × 15 µm and the scan rate was 0.3 Hz. The scanning was performed from the scale base to the apex only (see Supplementary Figure S2).

## 3. Results

### 3.1. Surface Observations and Analysis of Firebrat Scales

**Figure 1:**
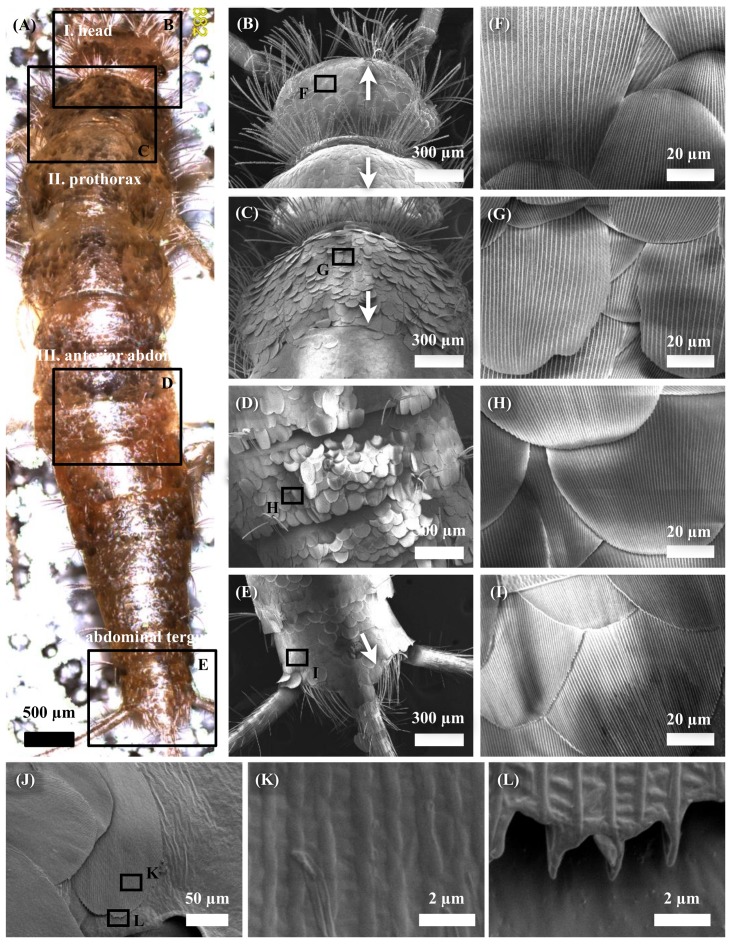
Surface observations of a firebrat by using a light microscope and FE-SEM. (**A**) Light microscopy image of an adult male firebrat. (**B**–**E**) FE-SEM images of firebrat surfaces for confirming alignments of scales on the firebrat integument: (**B**) head (I); (**C**) prothorax (II); (**D**) anterior abdominal region (III); and (**E**) 9th abdominal tergum (V). White arrows indicate the growth directions of scales. (**F**–**I**) Higher magnification images from (**B**–**E**), respectively. (**J**–**L**) FE-SEM images of backside of the scales. (**K**,**L**) Higher magnification images from (**J**).

Figure 1 shows the light microscope and FE-SEM images of the body surface of an adult firebrat (male, body length of 7.24 mm). The body surface of firebrats is densely covered with procumbent scales. Scales on the head are oriented transversally to the body axis (white arrows in Figure 1B–E). FE-SEM observations also revealed that the scale outer face have periodic groove structures (Figure 1F–I). These grooves were formed on only the face, and the backside of the scale has ladder-like structures of lower height and is regarded as almost flat when compared with grooves on the outer face (Figure 1J–L). The groove wavelengths of the firebrat scales appeared to vary between scales on the anterior regions of the body, particularly around the head, although the groove wavelengths are almost uniform within a scale (Figure 1F).

**Figure 2:**
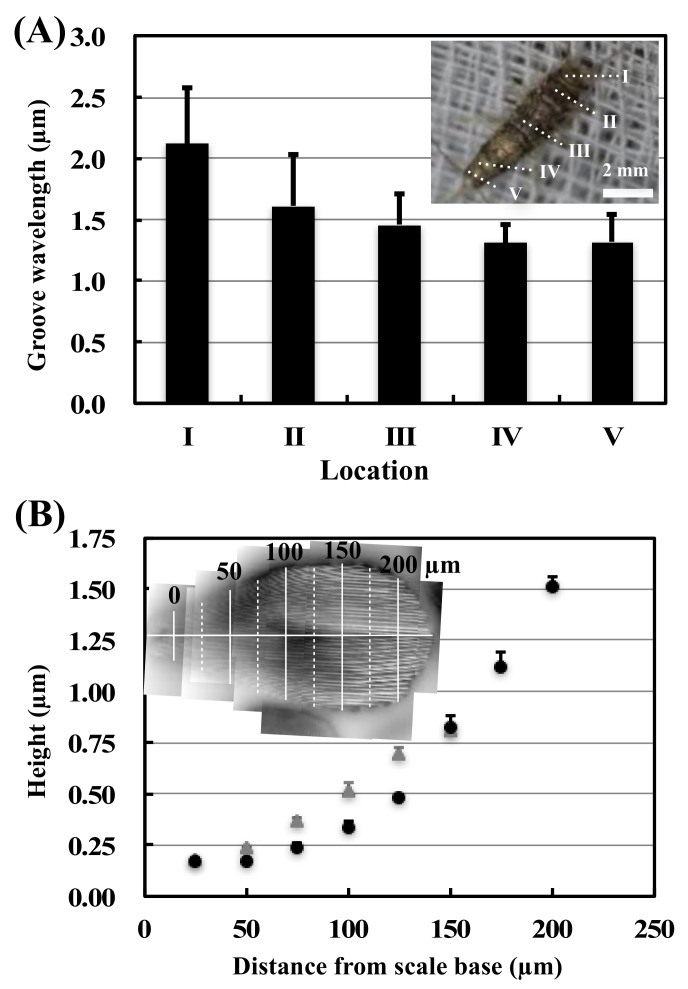
Graphs of structural variations of firebrat scales. (**A**) Groove wavelengths of firebrat scales (mean ± standard deviation) for the different body regions: head (I); pronotum (II); metanotum (III); 8th abdominal tergum (IV); and 9th abdominal tergum (V). (**B**) Groove height measured from the scale base to the apex. Data shown present the average height of three scales with a groove wavelength of ca. 3.5 µm (black) and three scales with a groove wavelength of ca. 2.0 µm (gray). Error bars represent the standard deviation. The inset shows a stitched image of AFM topographies. White lines indicate the positions of the grooves’ measuring height.

To study the differences in groove wavelengths more precisely, we analyzed the groove wavelengths by periodicity detection using autocorrelation. It was revealed that the groove wavelengths and their standard deviations are clearly smaller from the firebrat’s head to the tail. Also, groove wavelengths become smaller to the tail (Figure 2A). Figure 2B shows the graphs of groove height from the scale base to the apex of six scales, with groove wavelengths of ca. 3.5 µm (in black) and ca. 2.0 µm (in gray), as measured by AFM. According to the graph, the groove heights increase almost proportionally with to the distance from scale base to apex. Thus, the groove heights are also varying within a scale. 

### 3.2. Surface Chemical and Wettability Analysis of Firebrat Scales

**Figure 3:**
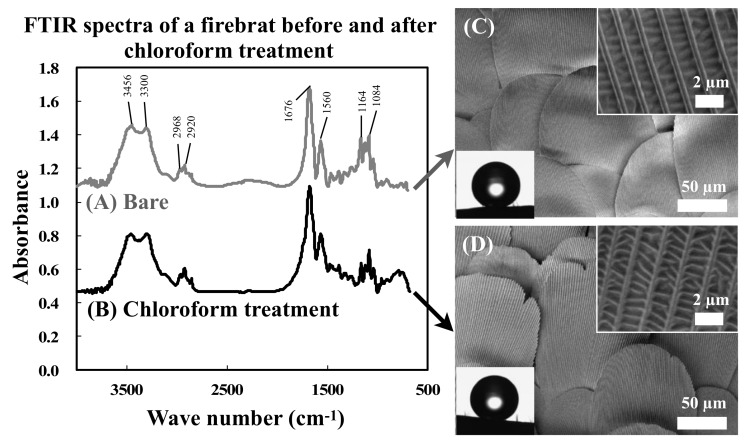
Surface analysis of firebrat scale surface before and after chloroform treatment. (**A**,**B**) FTIR spectra and (**C**,**D**) FE-SEM images and photographs of water droplets on the firebrat dorsal surface. (**A**,**C**) Bare surface and (**B**,**D**) chloroform-treated surface.

If firebrat surfaces are covered by a thick waxy compound layer, surface conditions would be changed during friction force measurements. To address this issue, we analyzed the surface chemistry and wettability of scales. Figure 3 shows FTIR spectra, FE-SEM images, and photographs of water droplets on scales before and after immersion in CHCl_3_ for 10 min. In the FTIR spectra, the peaks that are attributed to the vibration modes of asymmetric stretching of O–H (3456 cm^−1^), amide A bands stretching from N–H stretching (3300 cm^−1^), asymmetric stretching of COCH_3_ (2968 cm^−1^), stretching of C–H (2920 cm^−1^), stretching of C=O (1676 cm^−1^), bending of N–H (1560 cm^−1^), stretching of C–O (1164 cm^−1^), and symmetric phosphate (PO_2_^−^) stretching (1084 cm^−1^) were detected [29,30]. Assignments of the relevant bands of FTIR spectra of the firebrat surfaces before and after CHCl_3_ treatment are shown in Table 1. The water contact angles of the firebrat surfaces before and after CHCl_3_ treatment were 157.9 ± 3.0° and 153.5 ± 2.8°. Differences of water contact angles were caused by a little shape change of the firebrat by CHCl_3_ treatment (inner fluid was extracted and the body flattened).

### 3.3. Friction Measurements on a Body Surface–Needle Probe

**Figure 4:**
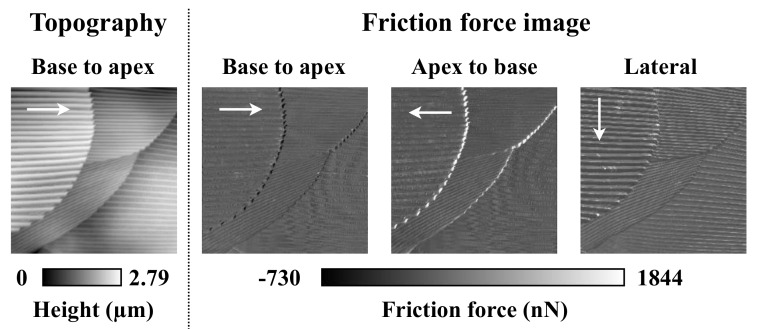
Topography and friction force images obtained by AFM with a needle probe. White arrows show the scanning direction. The body part measured was the dorsal pronotum, and the scanning area was 50 µm × 50 µm.

Friction forces on overlapped scales were measured using AFM with a needle-type probe along three scanning directions. According to the friction force images in Figure 4, friction anisotropy dependencies of scanning directions at the boundaries of overlapped scales appeared. At the scale boundary, the friction forces were markedly reduced when examined along the scale base to apex scanning direction. In contrast, friction forces were increased at the boundary along the scale apex to base scanning direction. Moreover, friction forces were increased at the top of the grooves in the case of lateral scanning. Groove structures appear to act as projections; however, this is unconcerned for firebrats, as they cannot move sideways.

### 3.4. Friction Measurements on a Body Surface–Colloidal Probe

**Figure 5:**
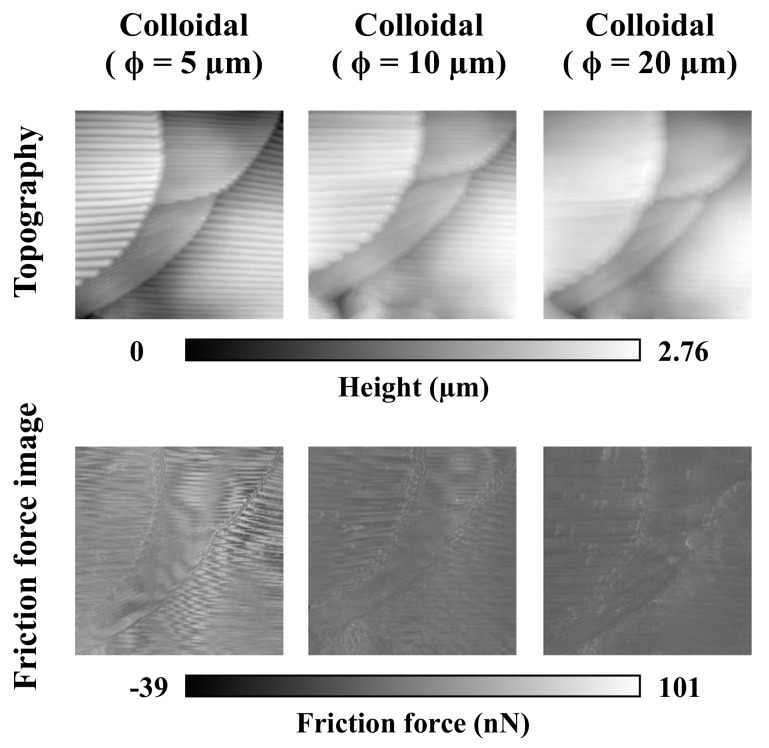
Topographies and friction force images obtained by AFM with three types of colloidal probe. Scanning direction was scale base to apex only. The body part measured was the pronotum.

We next focused on the groove structures on the firebrat scale surface. Since we predicted that one of the properties of groove structures was friction reduction by reducing the contact area between scales and their surroundings, friction forces were measured by using AFM with three types of colloidal probes (with diameters of 5, 10, 20 µm), which diameters are larger than the groove wavelengths. Consistent with the AFM measurements (Figure 5), the contact area was decreased with increasing probe diameter becoming difficult to distinguish small groove structures in topographies. When indenters became larger, the contact areas of the indenters were regarded as a plane surface and they negated the effects of the groove structures. Similarly, friction forces were reduced with increasing probe diameter. These findings suggest that friction force is proportional to the contact area.

### 3.5. Friction Measurements within a Scale

**Figure 6:**
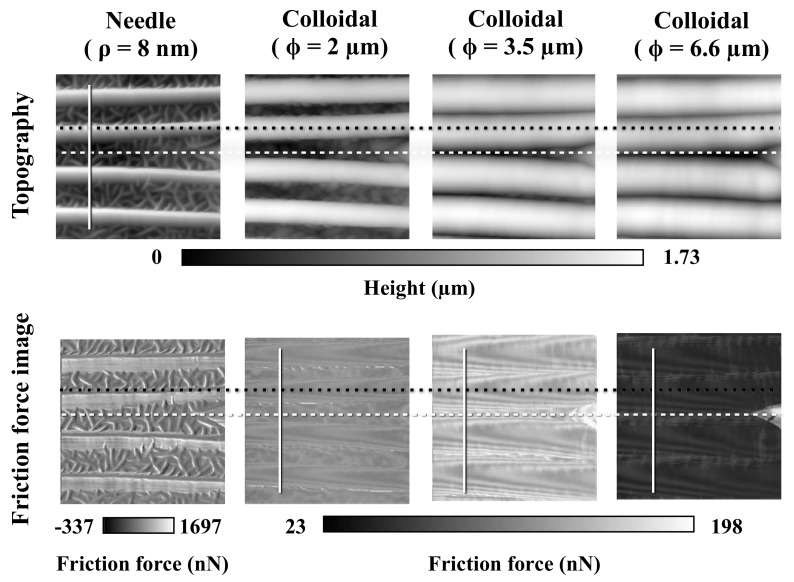
Friction force images obtained by AFM. Black dotted lines indicate the top of the grooves and white broken lines indicate the bottom of grooves. White lines are sampling points of the height profile and the friction force as shown in Figure 7. The scanning direction was the same (left to right), and the scanning area was 15 µm square. Within this figure, same scale spot was measured by four different probes.

Figure 6 shows the topographies and friction force images obtained by AFM with a needle probe and colloidal probes with diameters of 2.0, 3.5, and 6.6 µm. In this case, scales with a groove wavelength of ca. 3.5 µm were selected for these measurements, so the diameters of colloidal probes are almost the same size of the groove wavelength. When comparing topographies, the colloidal probes with larger diameters were not able to reach the groove bottom. Friction forces were the largest for the colloidal probes of 3.5 µm in diameter. According to these friction measurements, there is a relationship between groove wavelength and colloidal probes when their sizes are similar; friction forces were not simply becoming larger with decreasing diameters of colloidal probes, as shown in Figure 4.

## 4. Discussion

### 4.1. Observations and Analysis of Firebrat Scales

According to the FE-SEM observations and analysis, firebrats are densely covered with scales with periodic groove structures on the outer face side (Figure 1K) and are almost flat on the backside (Figure 1L), and only scales on the head are oriented transversally to the body axis (Figure 1). The groove wavelength of scales differs between scales on the anterior regions of the body, although the groove wavelengths are almost uniform within a scale. The wavelengths are becoming smaller and uniform toward the tail (Figure 2A). Furthermore, groove heights are almost proportional to the distance from the scale base to the apex, in such a way that the groove heights also differ within a scale (Figure 2B). When comparing the FTIR spectra, two FTIR spectra appeared mostly unchanged. Furthermore, water contact angles before and after immersion in CHCl_3_ were 157.9 ± 3.0° and 153.5 ± 2.8°, respectively. These results suggest that the surface conditions did not change after CHCl_3_ treatment. Based on this observation, we assumed that the surface of firebrat scales is not covered by waxy compound layers that greatly influence for the friction force measurements by changing surface morphologies and, thus, we measured the friction force directly (in this study, we did not consider the thin layer of epicuticular grease nor the possibility of the existence of a molecular boundary lubricant at the firebrat surfaces, such as in snake skin [31], because those are difficult to remove completely). Furthermore, if surface waxy compounds were completely removed, the surface conditions of wild-type firebrat would change failing to reproduce the actual friction properties of firebrat scales.

Some of the features of the firebrat scales remained elusive. Differences in scale growth direction at the firebrat’s head, which is against forward movement, appear to be easily supposed to interfere with the animal’s movement. It also remains unclear the function of the different groove structure on the scale surfaces, as well as the groove wavelength and height unevenness. Ordinary scales are considered to have various effects, such as the prevention of water loss, escape from enemies [32], protection against/reflection of ultraviolet radiation [33], and coloring [34], but we speculate that firebrat scales might have also evolved for reducing friction to reduce wear and injury of their body, because firebrats temporary hide in narrow spaces [35] and the body surface is frequently attrited by surrounding surfaces [36]. This latter aspect has not been considered so far for firebrats. However, the literature describes friction force reduction by similar groove structures in an engine piston [37,38]. In order to address the friction properties of the firebrat scales, we measured the friction forces on the scale surfaces using AFM. 

### 4.2. Meaning of Scale Growth Directions

To investigate the meaning of scale growth orientations, friction forces on overlapped scales were measured using AFM with a needle-type probe and three kinds of scanning directions (Figure 4). As a result, the friction forces were larger or smaller at the scale boundaries depending on the scanning directions. This suggests that scale orientation on the prothoracic regions or thoracic and abdominal regions prevents sticking during forward movement into a narrow space (see Supplementary Video S2). Meanwhile, we inferred that firebrats use “high friction” that is generated at the head scale apexes (edges), where the growth directions are against the forward movement, with the mechanosensor to determine whether they can enter small spaces (the head is narrower than the prothoracic region). Furthermore, firebrats sometimes put their head into the narrow space when they search for food. In that case, if scale orientation on the head is the same to the body axis, firebrats head would be fixed and could not pull out (see Supplementary Video S2).

### 4.3. Assumed Effect of Groove Structures on Scales

We measured friction forces by AFM with three different size of colloidal probes to study the effects of groove structures on scales. In Figure 5, the topographies and friction force images indicate that firebrat use scale groove structures for the reduction of friction force by reducing contact area to the surroundings. In fact, the firebrat’s scale backside, which is always in contact with a scale face, is nearly flat. These results suggest that the firebrat utilizes scales with groove structures for a reduction of friction. 

### 4.4. Considerations about Groove Wavelengths and Heights Unevenness

Generally, the groove wavelengths on the scales are the same over the body of insects, such as butterflies. In butterflies, uniform groove wavelengths on scales are used for the generation of optical properties referred to as structural color [34]. We speculated that this groove wavelength unevenness may have frictional properties, because firebrats are nocturnally active and they live in narrow dark spaces, such as piled papers, in the daytime, therefore optical properties appear not to be necessary. Moreover, the standard deviations of the groove wavelengths on the head that frequently come into contact with their surroundings (see Supplementary Video S3) are large, whereas those on parts that have little contact, such as the tail, are small. This suggests that the firebrat has evolved uneven groove wavelengths for a specific purpose. 

**Figure 7:**
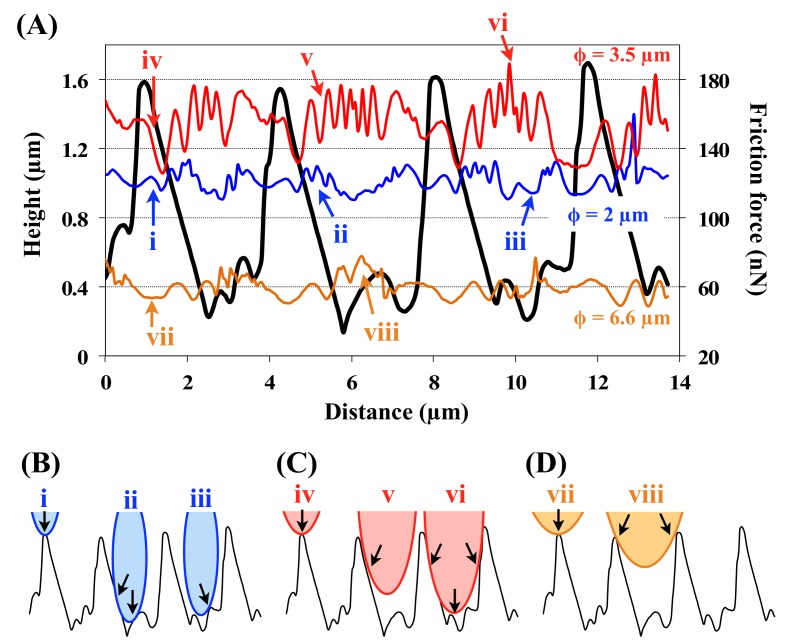
Graph and schematic illustrations showing the relationship between groove structures, friction force and colloidal probes. (**A**) Height profiles of the groove structures (black line) and friction forces (colored lines). The data were selected from the white lines on the friction force images in Figure 4. (**B**–**D**) Schematic of the relationship between groove structures and the (**B**) 2, (**C**) 3.5, and (**D**) 6.6 µm diameter colloidal probes. Black arrows indicate the contact area between groove structures and colloidal probes.

To investigate the effects of groove wavelength unevenness, friction forces on 3.5 µm groove wavelength were measured by using AFM with three different diameter colloidal probes, where the sizes have similar dimensions to the scale groove wavelength (Figure 6). As a result, the friction forces were not simply becoming larger with decreasing diameters of colloidal probes, but rather, the friction forces were larger when a diameter of a colloidal probe and a groove wavelength were same. To estimate this relationship between groove structures and colloidal probes, the height profile of groove structures and friction forces were analyzed (selected positions are shown in Figure 6 as white lines). Figure 7A shows the height profile of groove structures (black line) that was obtained from the topography and friction forces (colored lines) obtained from the friction force images in Figure 5, respectively. Figure 7B–D shows the schematic illustrations of the groove structures and colloidal probes of three different diameters, respectively. In the case of the 2 µm diameter probe, the friction force was larger at the bottom and sides of the grooves (displayed as “b” in Figure 7A,B) as the colloidal probe made contact with two surfaces. In the case of the 3.5 µm diameter probe, which was similar in size to the groove wavelengths, the friction forces were largest and they became larger toward the center of the grooves (displayed as “f” in Figure 7A,C). When the diameter of the colloidal probe was 6.6 µm, the friction forces were increased at the center of grooves for the same reason as described above. However, as the contact areas were small, the friction forces were smaller than those for the other probes. These results clearly suggest that an increment in contact area causes larger friction forces, such as in case of gecko or insect foot pads [39]. In particular, if the probe diameter and the groove wavelengths are the same size, the probe sticks in the grooves, leading to a larger friction force. The same trends were observed at 2 µm groove wavelength (see Supplementary Figure S3). According to these results, the groove structures are likely to influence friction, and inhomogeneous groove wavelengths may prevent high levels of friction with rough surfaces of specific size by preventing the fixation of all the scales.

## 5. Conclusions

We observed the body surface of firebrats, which is densely covered with scales, by FE-SEM and measured friction force by colloidal probe AFM as part of our investigation of the properties of the firebrat scales. FE-SEM observations and periodicity analysis of scale surface microgrooves revealed that the groove wavelengths differ by body part, although the groove wavelengths are almost uniform within each scale. AFM measurements suggested that firebrat scales have four properties about friction; one is the direction of scale growth on the prothoracic region or thoracic and abdominal regions. This prevents the firebrat from becoming lodged during movement in narrow spaces. The second one is the growth direction of the head scales. The head scales act as a mechanosensor to identify whether they can enter a narrow space. The third is the groove structures, which reduce contact area and lead the reduction of friction forces. The fourth property is the heterogeneous groove wavelengths. Due to changes in groove wavelength for each scale, it is difficult to fix the whole-body scales at a time. If some scales are stuck to something, then the firebrat can easily escape by removing only the trapped scale (scales molt and regenerate [40]). These are also suggested by the standard deviations of groove wavelengths, which are larger toward to the anterior portion of the body, which is frequently in contact with the environment, and smaller on the body parts, such as the tail, which have little external contact. From our results, we concluded the firebrat scale may have evolved for reducing friction. Based on this knowledge of firebrat scales, we will design general surface texturing for developing novel less-frictional surfaces that are applicable to a wide variety of industrial fields [10,41].

## Figures and Tables

**Table 1:** Assignments of the relevant bands of FTIR spectra of the firebrat surfaces before and after CHCl_3_ treatment.

Assignments	Wave Number (cm^−1^)	
	Before Chloroform Treatment	After Chloroform Treatment
ν (O–H)	3456	3450
ν (N–H)	3300	3300
ν (COCH_3_)	2968	2966
ν (C–H)	2920	2922
ν (C=O of *N*-acetyl group)	1678	1678
δ (N–H of *N*-acetyl group)	1560	1562

ν: Stretching; δ: Bending.

## Supplementary Materials

The following are available online at http://www.mdpi.com/2313-7673/4/1/2/s1, Figure S1: Photograph of a firebrat specimen used for AFM measurements (direct measurement of the friction forces of scales), Figure S2: Photographs of scales fixed on a silicon substrate for AFM measurements (detailed measurement of the friction forces of scales), Figure S3: Friction force images obtained by AFM, Table S1: Value data set for Figure 2A,B, Table S2: Information of the cantilevers used in Figure 4, Figure 5 and Figure 6, Video S1: Contact of the firebrat prothorax region during movement of narrow space, Video S2: Frequent contact of firebrat head to surroundings during search for food, Video S3: Contact of the firebrat head to surroundings when a firebrat tries to cross a gap.
