# Supplementary material for: The Friction Properties of Firebrat Scales
Source: Biomimetics (Basel). 2019 Jan 4;4(1):2. doi: 10.3390/biomimetics4010002 (PMC6477615; doi:10.3390/biomimetics4010002)
Supplement: Supplementary file 1 [file biomimetics-04-00002-s001.zip › biomimetics-362058-SI/biomimetics-362058-Supplementary.pdf]

Supplementary Materials: The Friction Properties of Firebrat Scales

Yuji Hirai [1,*], Naoto Okuda [1], Naoki Saito [2], Takahiro Ogawa [2], Ryuichiro Machida [3], Shûhei Nomura [4], Masahiro Ôhara [5], Miki Haseyama [2] and Masatsugu Shimomura [1]

[1] Chitose Institute of Science and Technology, Bibi758-65, Chitose 066-8655, Hokkaido, Japan; hi7liter1fire0b@gmail.com (N.O.); m-shimom@photon.chitose.ac.jp (M.S.)
[2] Graduate School of Information Science and Technology, Hokkaido University, N-14, W-9, Kita-ku, Sapporo 060-0814, Hokkaido, Japan; saito@lmd.ist.hokudai.ac.jp (N.S.); ogawa@lmd.ist.hokudai.ac.jp (T.O.); miki@ist.hokudai.ac.jp (M.H.)
[3] Sugadaira Research Station, Mountain Science Center, University of Tsukuba, Sugadaira Kogen, Ueda 386-2204, Nagano, Japan; machida@sugadaira.tsukuba.ac.jp
[4] Department of Zoology, National Museum of Nature and Science Amakubo 4-1-1, Tsukuba 305-0005, Ibaraki, Japan; nomura@kahaku.go.jp
[5] The Hokkaido University Museum, N 10, W8, Sapporo 060-0810, Hokkaido, Japan; ohara@museum.hokudai.ac.jp
* Correspondence: y-hirai@photon.chitose.ac.jp; Tel.: +81-123-27-6068

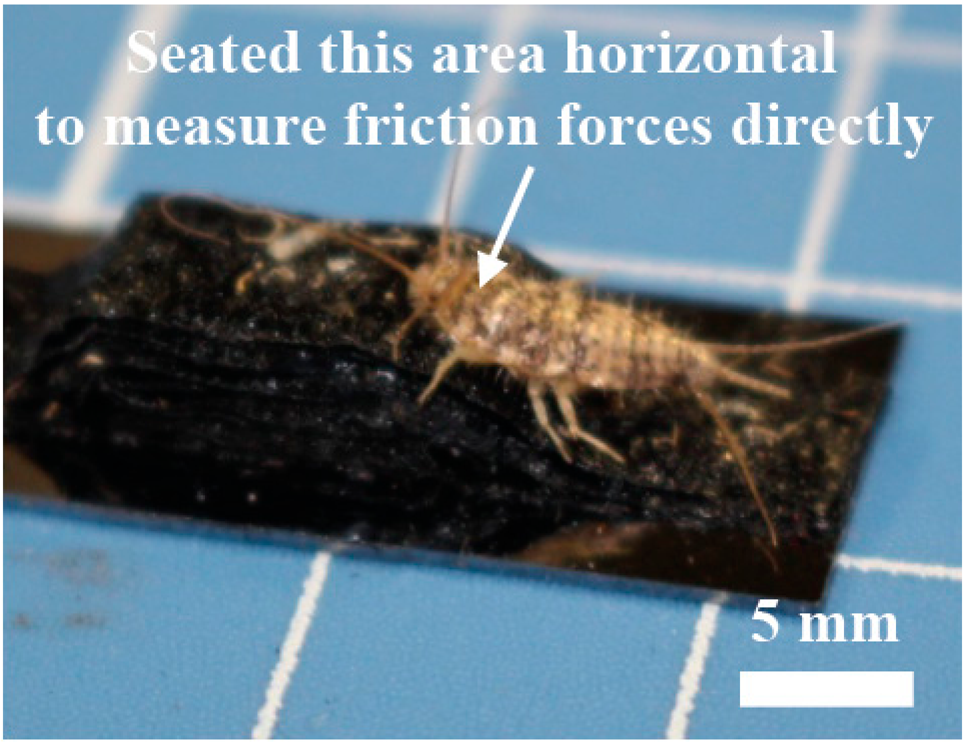

Figure S1. Photograph of a firebrat specimen used for AFM measurements (direct measurement of the friction forces of scales).

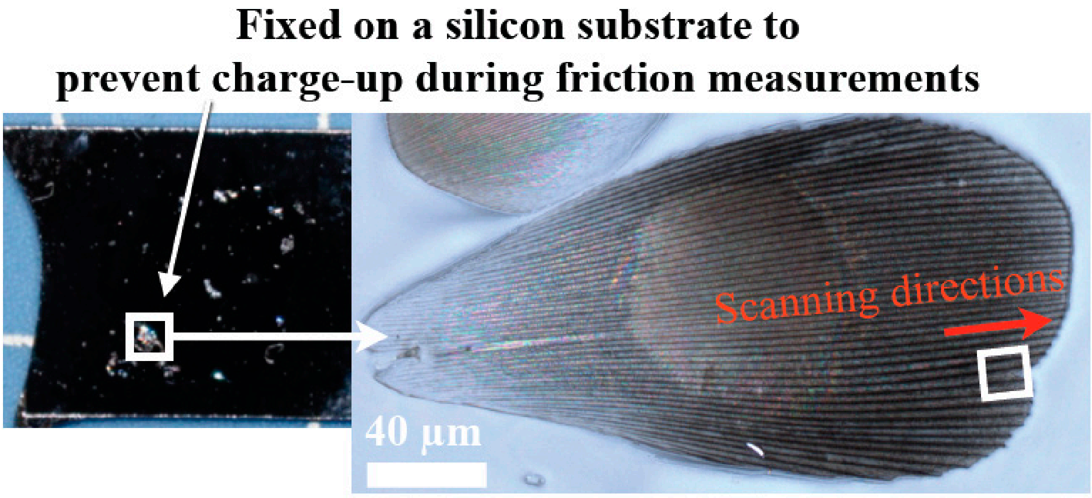

Figure S2. Photographs of scales fixed on a silicon substrate for AFM measurements (detailed measurement of the friction forces of scales).

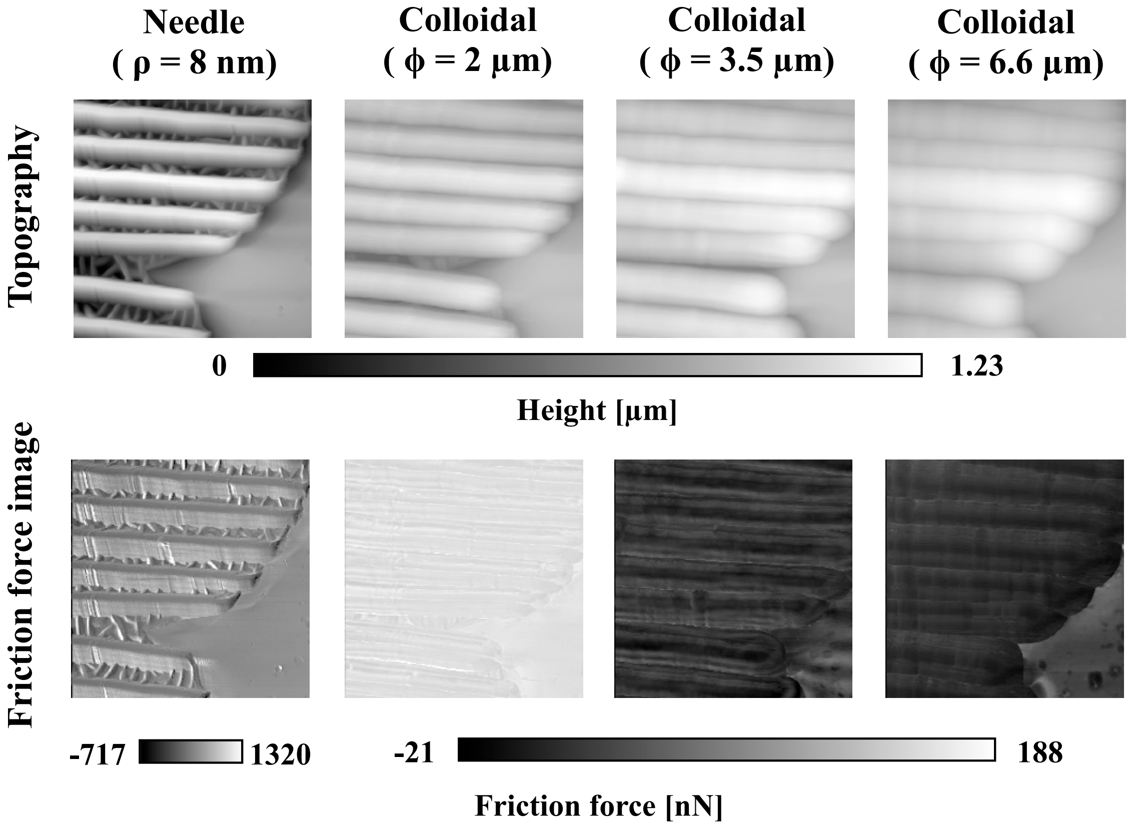

Figure S3. Friction force images obtained by AFM. A scale wavelength is ca. 2 μm. The scanning direction was the same (left to right), and the scanning area was 15 μm × 15 μm.

Table S1. (**a**) Value data set of Figure 2A.

**Location**	**Number of scales** [1]	**Average groove wavelength (μm)**	**± SD**
Head (I)	40	2.12	0.45
Prothorax (II)	100	1.61	0.42
Anterior abdominal (III)	80	1.46	0.25
8th abdominal tergum (IV)	35	1.31	0.15
9th abdominal tergum (V)	43	1.32	0.22

[1] Observable scale numbers from one firebrat by SEM (Figure 1).

Table S1. (**b**) Value data set of Figure 2B.

**Groove wavelength (μm)**	**Average groove height (μm)**								
	**Distance from the base (μm)**								
	**25**	**50**	**75**	**100**	**125**	**150**	**175**	**200**	**225**
2.0	0.163	0.256	0.400	0.511	0.626	0.780	-	-	-
2.0	0.178	0.234	0.377	0.595	0.767	0.806	(1.025)	-	-
2.0	0.189	0.213	0.322	0.445	0.689	0.834	(0.833)	-	-
3.5	0.174	0.144	0.198	0.287	0.426	0.827	1.065	1.455	(1.685)
3.5	0.211	0.193	0.244	0.307	0.503	0.700	0.989	1.460	(1.599)
3.5	0.139	0.188	0.282	0.414	0.509	0.944	1.303	1.625	-

Table S2. (**a**) Information of the cantilevers used in Figures 4 and 5.

	**Tip material**	***ρ*** **(nm)**	***ϕ*** **(µm)**	***L*** **(µm)**	***W*** **(µm)**	k_n **(N/m)**	k_l **(N/m)**
Needle probe	Silicon	8	-	224	23	2.30	235.74
Colloidal probe (5 µm)	Borosilicate	-	5	450	50	0.25	411.52
Colloidal probe (10 µm)	Borosilicate	-	10	450	50	0.22	122.44
Colloidal probe (20 µm)	Borosilicate	-	20	450	50	0.31	33.59

ρ: Tip curvature radius (needle type probe); *ϕ*: Tip diameter (colloidal probe); *L*: Cantilever's length; *W*: Width; k_n: Normal spring constant; k_l: Lateral spring constant.

Table S2. (**b**) Information of the cantilevers used in Figure 6.

	**Tip material**	***ρ*** **(nm)**	***ϕ*** **(µm)**	***L*** **(µm)**	***W*** **(µm)**	k_n **(N/m)**	k_l **(N/m)**
Needle probe	Silicon	8	-	228	23	2.60	277.11
Colloidal probe (2.0 µm)	SiO_2	-	2.0	450	50	0.22	1646.09
Colloidal probe (3.5 µm)	SiO_2	-	3.5	450	50	0.24	731.60
Colloidal probe (6.6 µm)	SiO_2	-	6.6	450	50	0.24	255.14

ρ: Tip curvature radius (needle type probe); *ϕ*: Tip diameter (colloidal probe); *L*: Cantilever's length; *W*: Width; k_n: Normal spring constant; k_l: Lateral spring constant.
